# Resveratrol and β-hydroxy-β-methylbutyric acid supplementation promotes ileal development and digestive function by altering microbial community abundance and metabolites in Tibetan sheep

**DOI:** 10.3389/fvets.2024.1470992

**Published:** 2024-12-11

**Authors:** Jiacheng Gan, Qiurong Ji, Quyangangmao Su, Shengzhen Hou, Linsheng Gui

**Affiliations:** College of Agriculture and Animal Husbandry, Qinghai University, Xining, China

**Keywords:** resveratrol, β-hydroxy-β-methylbutyric acid, Tibetan sheep, ileum, microorganism, metabolite

## Abstract

**Introduction:**

The effects of resveratrol (RES) and β-hydroxy-β-methylbutyric acid (HMB) on phenotype, immunity, digestive enzyme activity and short-chain fatty acids (SCFAs) contents in ileum of Tibetan sheep were investigated.

**Methods:**

A total of 120 two-month-old Tibetan sheep (15.5 ± 0.14 kg) were randomly allocated to 4 treatments: control group (basal diet), RES group (basal diet +1.5 g RES/d), HMB group (basal diet +1.25 g HMB/d), RES-HMB group (basal diet +1.5 g RES/d + 1.25 g HMB/d).

**Results:**

Results indicated that dietary RES and (or) HMB supplementation significantly improved the phenotype (mucosal thickness and villus width), SCFAs concentrations, and digestive enzymes (lipase, cellulase, and *α*-amylase) (*p* < 0.05). The relative abundance of *Brevibacillus*, *Clostridium sensu stricto 3*, and *Eubacterium hallii group* were increased, while the abundance of *Ruminococcus* and *Mogibacterium* were decreased (*p* < 0.05) in the RES-HMB group. The metabolic profiling indicated an increase in the differential metabolites (DMs) including L-arginine, butanoic acid, D-mannose, and retinol were increased in the RES-HMB group (*p* < 0.05).

**Discussion:**

In summary, our results suggested that RES and (or) HMB supplementation improved SCFAs concentration by up-regulating the microbial community abundance (*Brevibacillus*, *Clostridium sensu stricto 3*, and *Eubacterium hallii group*) and metabolism (L-arginine, butanoic acid, D-mannose, and retinol), thus contributing to ileal morphology and digestive enzyme activity. These findings may provides a novel reference for the nutritional regulation to improve the production of Tibetan sheep.

## 1 Introduction

Tibetan sheep (*Ovis aries*) is an important animal species living above 3,000 m on the Qinghai-Tibet Plateau (QTP) and a valuable economic resource for indigenous herders. Over a long period of natural selection, Tibetan sheep have now adapted to the QTP environment, which is characterized by low temperature, hypoxia, nutritional deficiency and strong ultraviolet radiation (1). Currently, approximately 14 million Tibetan sheep thrive well in the Qinghai province of China, providing meat, wool, skin, and other means of living (2). The small intestine is a complex ecosystem that aids in nutrient absorption and neurotransmitter production (3), both of which are essential aspects in maintaining general health (4). The ileum is a part of the small intestine. Previous studies suggested that the ileal microbiota, together with its metabolites modulated the metabolism, immune system, and health of the host (5). Therefore, understanding the function of the ileal microbiota and its metabolites could further our understanding of the absorption mechanism in the intestinal tract.

Several previous studies have indicated that dietary exogenous supplements including sauropus androgynus (6), anthocyanin-rich black cane (7), and quercetin (8) improved the ruminant health and economic character (9). Resveratrol (RES) is a non-flavonoid polyphenol compound, which is mainly extracted from natural plants (i.e., knotcane, peanut and apple). Previous research suggested it is involved in various biological functions including antioxidant stress (10), inflammatory response (11), and glucolipid metabolism (12). As an intermediate product of the leucine metabolic pathway, β-hydroxy-β-methylbutyric acid (HMB) participates in the protein synthesis (13), mitochondrial function (14), and fat deposition (15). Dietary supplementation with RES and HMB promoted the hepatic antioxidant capacity, immune response, and glycolytic activity through modulating the transcriptome and metabolome (16).

Our previous study showed that supplementing the diet with RES and HMB improved butyrate concentration by regulating the microbial community (*Methanobrevibacter*, *Actinobacteriota*, and *Bacillus*) and metabolism (*α*-ketoglutarate, succinic semialdehyde, and diacetyl), thus contributing to jejunal morphology, antioxidant capacity, immune response, digestive enzyme activity, and barrier function. Based on the structure of the small intestine (duodenum, jejunum and ileum), we hypothesized that supplementation with RES and HMB could affect the ileal function in Tibetan sheep. Therefore, the aims of present study to explore the mechanism of RES and HMB alone or in combination on the phenotype, immunity, digestive enzyme activity and short-chain fatty acids (SCFAs) contents in ileum of Tibetan sheep by 16s RNA gene sequencing and non-targeted metabolomics.

## 2 Materials and methods

### 2.1 Ethical statement

The protocol and methodology of the present study were approved by the Institution of Animal Care and Use Committee at Qinghai University, China (Xining, China; Permit No. QUA-2020-0709).

### 2.2 Animal experiments and sample collection

The experiment was conducted in Jinzang Pasture, Haiyan County, Haibei Tibetan Autonomous Prefecture, Qinghai Province. A total of 120 two-month-old healthy male Tibetan sheep with similar body conditions were selected and randomly divided into 4 groups with 30 sheep per group, including 1 control group (basal diet, C group) and 3 experimental groups: basal diet + RES (1.5 g/ day, RES group), basal diet + HMB (1.25 g/ day, HMB group), basal diet + RES (1.5 g/ day) + HMB (1.25 g/ day, RES-HMB group). The RES (purity > 99%) used in this experiment was purchased from Xi’an Grass Plant Technology Co., Ltd. (Xi’an, China). HMB (purity > 99%) was purchased from TSI Group Co., Ltd. (Shanghai, China). According to the additive dose, both RES and HMB were weighed and mixed evenly in the basal diet evenly before feeding. The pre-test period was 10 days, and the trial period was 90 days. The diet consisted of concentrate supplement and roughage with a ratio of 7:3. Roughage consisted of oat green hay and oat silage (1:1 dry matter mixture). The composition and nutritional levels of the experimental basal diet are shown in Table 1.

**Table 1:** Ingredients and nutrient levels of the experimental basal diet (on a dry matter basis).

	Items	Content (%)
Ingredient (%)	Corn	51.50
	Soybean meal	2.00
	Rapeseed meal	12.80
	Cottonseed meal	2.00
	Palm meal	25.00
	NaCl	1.00
	Lime stone	1.00
	Baking soda	0.10
	Premix^a^	4.60
	Total	100.00
Nutrient levels^b^	DE (MJ·kg^−1^)	12.71
	Crude protein	14.27
	Ether extract	3.29
	Crude fiber	11.64
	Neutral deterzent fiber	26.70
	Acid detereent fiber	19.97
	Ca	0.86
	P	0.40

a Premix diets per kg provide: Cu 18.00 mg, Fe 66 mg, Zn 30 mg, Mn 48 mg, Se 0.36 mg, I 0.60 mg, Co 0.24 mg, VA 24000 IU, VD 4800 IU, VE 48 IU.

b The digestible energy was calculated and the rest were measured.

At the end of the experiment, 24 Tibetan lambs (*n* = 6 per treatment) were slaughtered at a commercial slaughterhouse. Firstly, the terminal ileum were ligated with cotton thread to avoid loss of contents. The ileal samples (approximately 3 × 3 cm) were flushed with ice-cold phosphate-buffered saline (PBS) and then immediately dipped in 4% paraformaldehyde and 2.5% glutaraldehyde for histomorphometric microscopy analysis. Synchronously, the ileal content were collected in frozen pipe and then stored at −80°C for further analysis.

### 2.3 Measurement indicators and methods

#### 2.3.1 Ileal morphology

An appropriate amount of the ileal tissues was stored in fixed solution for 6–8 h. Next, the tissue samples were washed, dehydrated with an alcohol gradient, transparentized, waxed, embedded, sliced, and analyzed with hematoxylin & eosin (H&E) staining. The villus height, villus width, crypt depth, mucosal thickness, muscular thickness, and villus height/crypt depth (V/C) ratio were measured using Image-Pro Plus 6.0 software (Media Cybernetics, Bethesda, MD, United States).

#### 2.3.2 Enzyme-linked immunosorbent assay

The ileal contents were centrifuged (2,500× g) for 15 min at 4°C. The indicators of supernatant including immune and digestive enzyme were measured using the enzyme-linked immunosorbent assay kit (Enzyme Immunity Industry Co., Ltd., Nanjing, China). The immune indices were immunoglobulin A (IgA), IgG, IgM, tumor necrosis factor-*α* (TNF-α), interleukin-1β (IL-1β), and interleukin-6 (IL-6). while the digestive enzymes were lipase, trypsin, chymotrypsin, cellulase, and α-amylase.

#### 2.3.3 Determination of SCFAs contents

SCFAs in ileal contents, including acetic acid, propionic acid, butyric acid and isobutyric acid, were determined by gas chromatography according to the method described in reference (17). The content of SCFAs was calculated by standard curve using external standard method.

#### 2.3.4 Analysis of ileal microorganisms by 16S rRNA

The HiPure Stool DNA Kits (Magen, Guangzhou, China) was used to isolate bacterial DNA from ileal contents. The concentration of DNA was measured using a NanoDrop 2000 microspectrophotometer (Thermo Fisher Technologies, United States) and the quality of DNA was determined by agarose gel electrophoresis. Polymerase chain reaction (PCR) was used to amplify the V3-V4 hypervariable region of 16S rDNA gene using the forward primer 341F (5′-CCTACGGGNGGCWGCAG-3′) and reverse primer 806R (5′-GGACTACHVGGGTATCTAAT-3′). PCR amplification procedures refer to reference (18). After purification of PCR amplification products, Qubit 3.0 (Company New England Biolabs, United States) was used to detect the concentration of the products in Illumina NovaSeq 6000 (Illumina Inc., United States) on sequencing.

The original microbial sequences were processed using Trimmomatic (version 0.35) and FLASH (version 1.2.11) software. The sequences were clustered into operational taxonomic units (OTUs) with similarity ≥97% using Vsearch (version 2.4.2) software. All representative sequences for each OTU were selected using QME and entered into the Silva database (version 138) for comparison and annotation using RDP classifier (version 2.2) software.

#### 2.3.5 Ileal metabolomics analysis

The ileum contents (60 mg) was taken into a 1.5 mL EP tube and mixed with 20 μL standard solution (0.3 mg/mL *L*-2-chloro-phenylalanine, methanol) and 360 μL methanol: water mixture (4:1, volume ratio), then left for 2 min at −20°C and ground (60 Hz, 2 min); 200 μL chloroform and 400 μL water were added, swirled for 2 min, and ultrasonic extraction was performed in ice water bath for 30 min. Rest at −20°C for 30 min and centrifuge for 10 min (13,000 r/min, 4°C). Take 150 mL of the supernatant in a glass derived vial and dry it in a freeze concentrate centrifugal dryer. The quality control (QC) sample was prepared by mixing the extract of all samples in equal volume, adding 80 μL methoxyamine hydrochloride pyridine solution (15 mg/mL), swirling for 2 min, and incubating in an oscillating incubator at 37°C for 90 min, and then performing oximation reaction. Then, 50 μL bis (trimethylsilyl) fluoroacetamide (containing 1% trimethylchlorosilane), 20 μL n-hexane and 10 μL internal standard were added. After vortex oscillation for 2 min, the reaction was carried out at 70°C for 60 min and left at room temperature for 30 min. Metabolomics analysis was performed by gas chromatography–mass spectrometry (GC–MS).

### 2.4 Statistical analysis

Kruskal-Wallis H test was used to analyze the difference of *α* index among sample groups. Unweighted Unifrac distance metric is used for princi-pal coordinates analysis (PCoA). Differences in diversity among sample habitats were assessed by A-donis (permutation multivariate analysis of variables); Univariate analysis of variance was used to determine the differences between groups. Student’s *T* test was used to analyze and screen the metabolites with significant differences between groups [projected importance of variables (VIP) > 1, *p* < 0.05], and the selected metabolites were enriched using the Kyoto Encyclopedia of Genes and Genomes (KEGG) database.

Experimental data were analyzed by one-way ANOVA module in the SPSS 20.0 software package, and the Duncan method was used for multiple comparisons for those with significant differences. All data are expressed as standard error of mean (SEM), and *p* < 0.05 was considered to indicate statistical significance.

## 3 Results

### 3.1 Ileum histology

**Figure 1:**
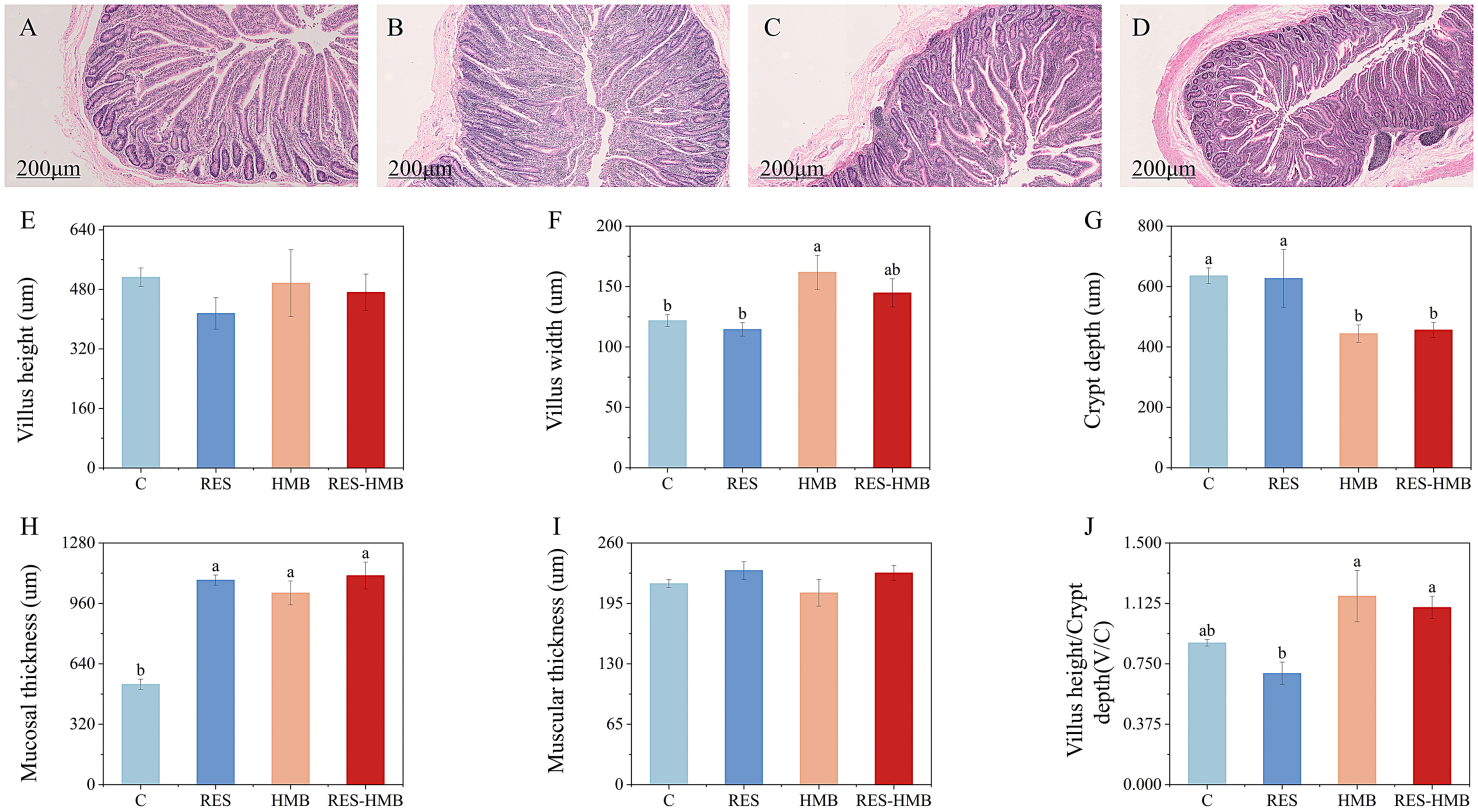
**(A–D)** Section of ileal tissue. **(A)** C group. **(B)** RES addition group. **(C)** HMB addition group. **(D)** RES-HMB addition group. H&E staining, 100× magnification. **(E–J)** Effects of RES or HMB on the morphology and development of ileal in Tibetan sheep. In the same figure, no letter or the same letter superscript indicates no significant difference (*p* > 0.05), while different lowercase letters superscript indicates significant differences (*p* < 0.05). The same below.

H&E sections revealed the morphological alternations in the ileum of Tibetan sheep (Figures 1A–D). Compared to the C and RES groups, HMB group exhibited a significant increased villus width (*p* < 0.05, Figure 1F). Whereas the villus width in RES group was significantly increased than that of C group and RES-HMB group (*p* < 0.05, Figure 1G). All treatments increased the muscular thickness (*p* < 0.05, Figure 1H). No significant difference was observed in villi height, and muscular thickness (Figures 1E,I, *p* > 0.05).

### 3.2 Immune response indexes

The effects of RES and HMB on ileal immunity were presented in Table 2. There was no difference observed for IgM, IL-1β, and IL-6 of this experiment (*p* > 0.05). The content of IgG and TNF-*α* in HMB group was significantly increased than that in RES-HMB group (*p* < 0.05). Compared with other treatments, the TNF-α content in the RES-HMB group significantly decreased (*p* < 0.05).

**Table 2:** Effects of RES/HMB on the ileal immune indexes of Tibetan sheep.

Items	Groups				*p*-values
	C	RES	HMB	RES-HMB	
IgA (μg/mL)	0.49 ± 0.21^b^	2.84 ± 0.32^a^	1.70 ± 0.58^ab^	1.65 ± 0.34^ab^	0.011
IgG (μg/mL)	989.72 ± 69.07^b^	1034.63 ± 99.66^ab^	1367.22 ± 142.23^a^	1299.44 ± 40.47^ab^	0.036
IgM (μg/mL)	10.36 ± 1.12	9.03 ± 1.70	9.86 ± 1.72	10.79 ± 1.66	0.877
TNF-α (ng/L)	1305.22 ± 8.01^a^	1289.67 ± 54.60^a^	1155.00 ± 30.92^a^	953.00 ± 51.77^b^	0.001
IL-1β (ng/L)	128.60 ± 12.69	114.58 ± 6.64	143.29 ± 10.87	130.05 ± 13.99	0.451
IL-6 (ng/L)	56.31 ± 6.36	59.61 ± 2.19	52.27 ± 12.23	52.86 ± 4.69	0.928

In the same row, values with no letter or the same letter superscripts mean no significant difference (*P* > 0.05), while with different small letter superscripts mean significant difference (*P* < 0.05).

### 3.3 Digestive enzyme activity of ileal contents

The effects of RES and HMB on digestive enzyme activity in ileal contents were presented in Table 3. The activities of lipase, cellulase, and α-amylase in RES-HMB group were significantly increased than that of the C group (*p* < 0.05). No difference of chymotrypsin activity was observed in all treatments (*p* > 0.05).

**Table 3:** Effects of RES/HMB on digestive enzyme activity in ileal contents of Tibetan sheep.

Items	Groups				*p*-values
	C	RES	HMB	RES-HMB	
Lipase (ng/L)	159.62 ± 9.67^c^	204.36 ± 7.62^b^	242.50 ± 13.21^a^	256.28 ± 10.91^a^	0.001
Trypsin (ng/mL)	10.25 ± 1.04^b^	10.88 ± 0.27^ab^	13.76 ± 0.44^a^	12.92 ± 0.99^ab^	0.018
Chymotrypsin (ng/L)	236.67 ± 11.77	241.33 ± 10.70	234.67 ± 31.53	274.00 ± 17.93	0.397
Cellulase (ng/L)	78.72 ± 0.64^b^	89.10 ± 2.92^b^	106.17 ± 4.81^a^	115.00 ± 1.97^a^	0.001
α-amylase (μmol/L)	57.11 ± 4.17^b^	59.97 ± 3.73^ab^	72.99 ± 3.63^a^	73.33 ± 3.84^a^	0.010

In the same row, values with no letter or the same letter superscripts mean no significant difference (*P* > 0.05), while with different small letter superscripts mean significant difference (*P* < 0.05).

### 3.4 SCFAs concentration of ileal contents

As showed in Table 4, the concentration of propionic acid, isobutyric acid, and butyric acid in RES-HMB group were significantly increased than that in C group (*p* < 0.05). While the acetic acid/propionic acid were significantly increased in the C group than in other treatments (*p* < 0.05).

**Table 4:** Effects of RES/HMB on SCFA contents in ileum of Tibetan sheep (μg/g).

Items	Groups				*p*-values
	C	RES	HMB	RES-HMB	
Acetic acid	0.82 ± 0.09	0.67 ± 0.03	0.65 ± 0.01	0.67 ± 0.02	0.094
Propionic acid	0.07 ± 0.01^c^	0.09 ± 0.01^b^	0.10 ± 0.01^ab^	0.11 ± 0.01^a^	0.005
Isobutyric acid	0.02 ± 0.01^c^	0.03 ± 0.01^b^	0.05 ± 0.01^a^	0.05 ± 0.01^a^	0.001
Butyric acid	0.04 ± 0.01^b^	0.12 ± 0.01^a^	0.10 ± 0.02^a^	0.09 ± 0.01^a^	0.010
Acetic acid/propionic acid	11.50 ± 1.08^a^	7.48 ± 0.92^b^	6.53 ± 0.39^b^	6.01 ± 0.43^b^	0.004

In the same row, values with no letter or the same letter superscripts mean no significant difference (*P* > 0.05), while with different small letter superscripts mean significant difference (*P* < 0.05).

### 3.5 Ileal microorganism

#### 3.5.1 Richness and diversity of the ileal flora

**Figure 2:**
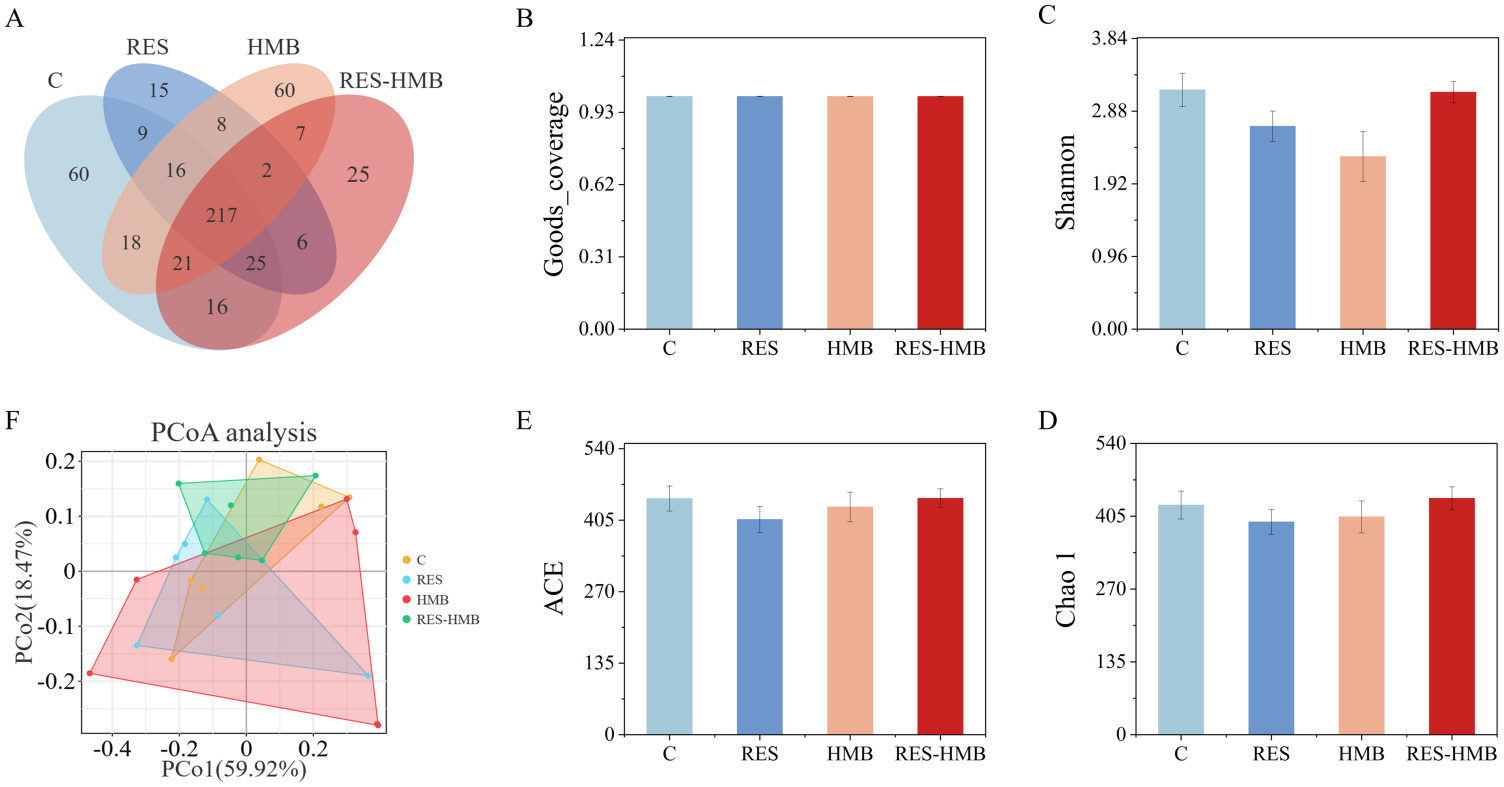
Richness and diversity of the ileal microflora. **(A)** OTUs Venn diagram for experimental groups and C group. **(B)** The Goods_coverage of the four groups. **(C–E)** Alpha diversity as presented by Shannon, Chao1, ACE in the ileum of Tibetan sheep among groups. **(F)** Principal coordinate analysis based on Bray-Curtis distance.

Microbial diversity was classified by OTUs based on sequence similarity. A total of 382, 298, 349, and 319 OTUs were determined in the C, RES, HMB, and RES-HMB groups, respectively. The ileum samples from the four treatment groups shared 217 OTUs, which accounted for 42.97% of the total valid sequences (Figure 2A). Alpha diversity analysis among the groups showed that the coverage of the four groups was greater than 0.997, indicating that the sequencing results could reflect the real situation of the samples (Figure 2B). The alpha diversity of the ileal microbiota was analyzed using Shannon, Chao1, and ACE indices (Figures 2C–E). No significant differences in the alpha diversity indexes were noted among all groups (*p >* 0.05), indicating that dietary supplementation of RES, HMB or both had no significant effect on ileal microbial diversity. PCoA based on Bray-Curtis distance was used to assess beta diversity of samples (Figure 2F). The points of each group were well separated from each other, and there were no obvious boundaries between the points of the experimental and C groups, indicating no significant changes in the intestinal flora composition of the experimental group.

#### 3.5.2 Composition of the ileal microbiota

**Figure 3:**
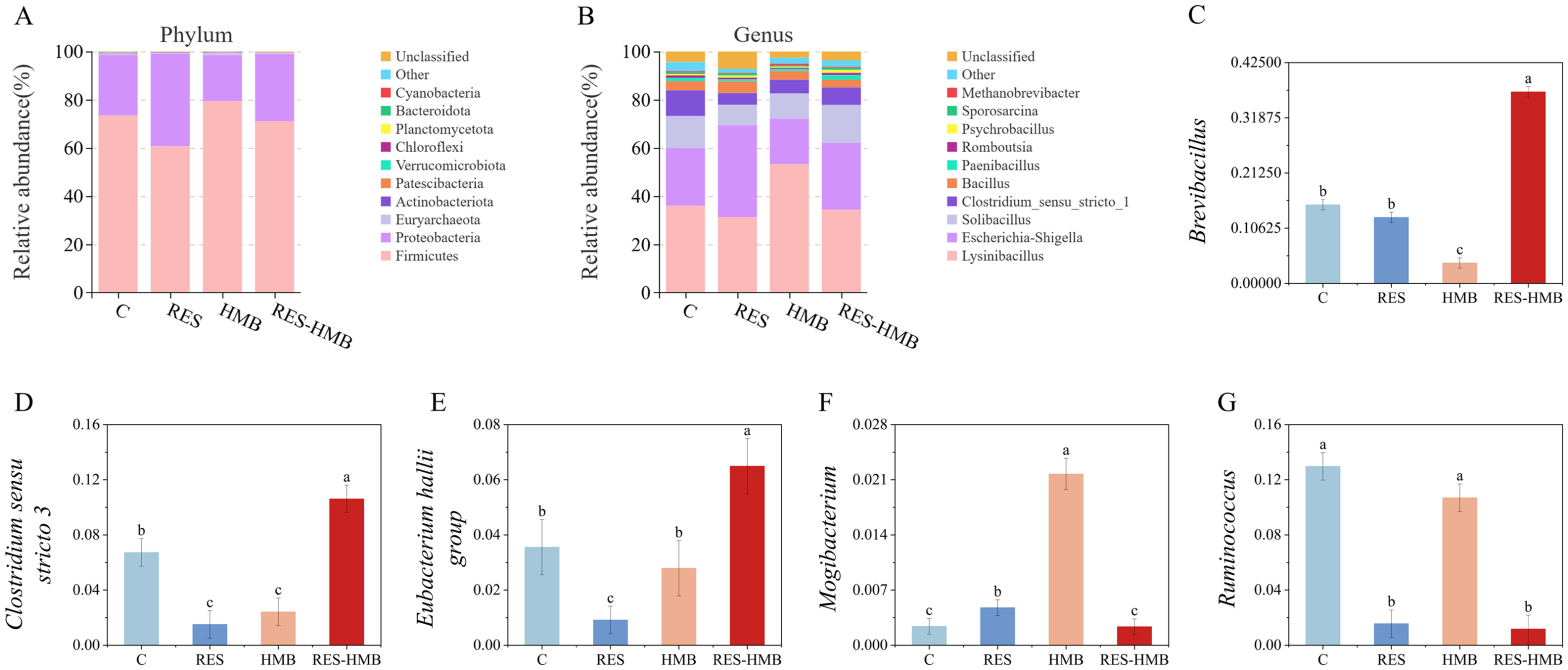
Composition of the ileal microbiota of Tibetan sheep at phylum **(A)** and genus **(B)** level. **(C–G)** Histogram showing differences in the contents of microflora taxonomic groups at the genus level.

To further evaluate the effects of different diets on ileal microflora, statistical analysis was performed at different microbial classification levels. At the phylum level (Figure 3A), there were 3 species of bacteria with relative abundance greater than 1% in the ileum of the four groups (C vs. RES vs. HMB vs. RES-HMB) of Tibetan sheep, including *Firmicutes* (73.42% vs. 60.64% vs. 79.53% vs. 71.10%), *Proteobacteria* (25.20% vs. 38.50% vs. 19.05% vs. 27.89%), and *Euryarchaeota* (0.52% vs. 0.30% vs. 0.79% vs. 0.42%). The HMB group had a higher relative ileal abundance of *Firmicutes* and *Bacteroidota* in ileum compared to C group (*p* < 0.05). The relative abundance of *Proteobacteria* was significantly higher in the ileal contents of the RES group than that of the C group (*p* < 0.05).

A total of 39 bacterial genera were identified at the genus level (Figure 3B). Irrespective of the dietary treatment, *Lysinibacillus* (31.22–53.26%), *Escherichia-Shigella* (18.88–38.23%), *Solibacillus* (8.51–15.72%), *Clostridium sensu stricto 1* (4.77–10.71%) and so on were the dominant genera comprising the ileal microbiota. Similarly, the relative abundance of *Escherichia-Shigella* in the ileum of RES group was greater than that in the C group (*p* < 0.05). The relative abundance of *Brevibacillus*, *Clostridium sensu stricto 3*, and *Eubacterium hallii group* in the RES-HMB group were higher than C group, whereas the relative abundance of *Ruminococcus* and *Mogibacterium* in the RES-HMB group was lower than that in C group (Figures 3C–G).

### 3.6 Ileal metabolomics

#### 3.6.1 Differences in ileal metabolome

**Figure 4:**
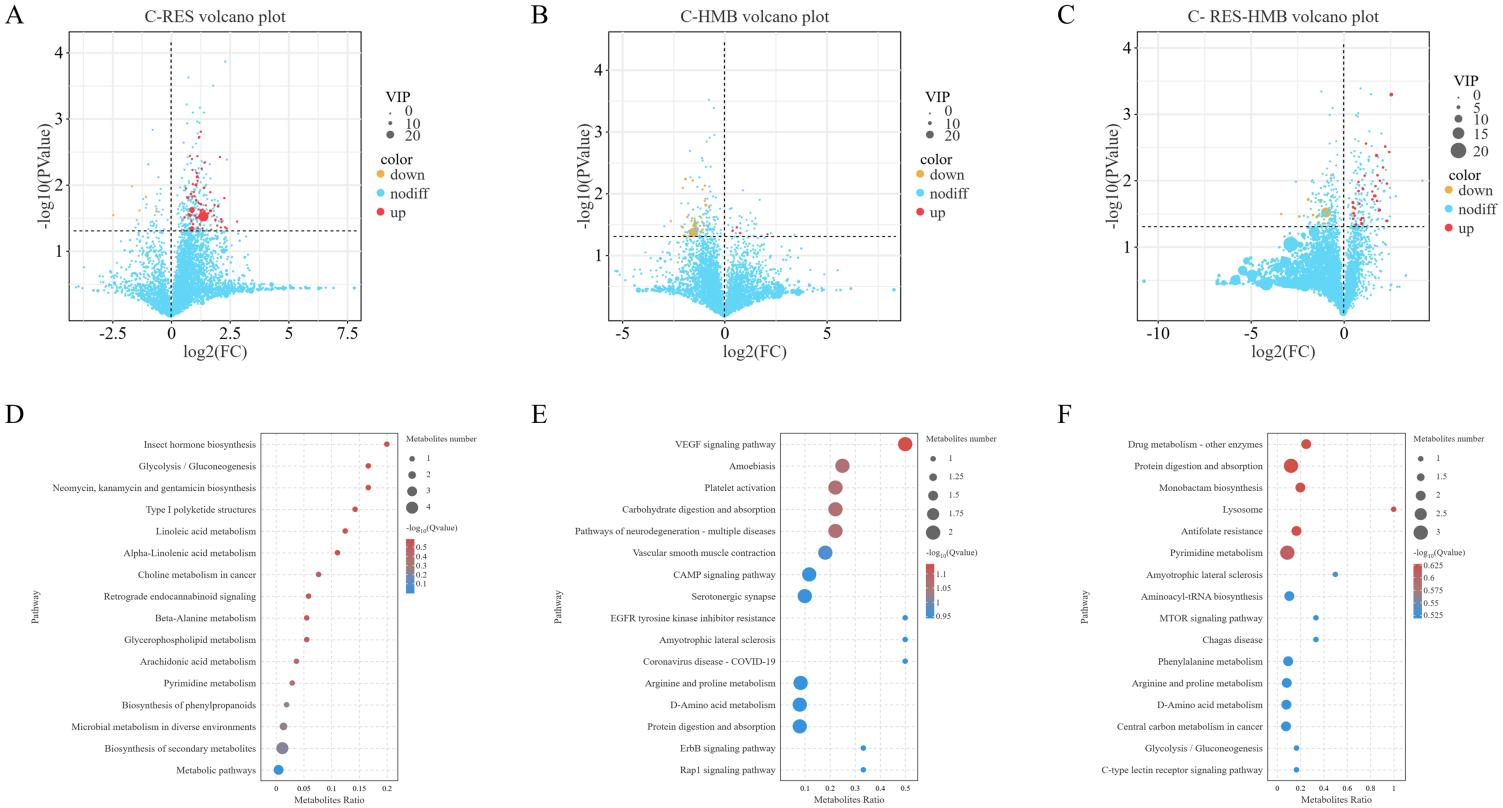
Differential metabolites and metabolic pathways between the experimental and control groups. **(A)** Volcano plot showing differential metabolites in the ileal tissues from the C-RES group. **(B)** Volcano plot showing differential metabolites in the ileal tissues from the C-HMB group. **(C)** Volcano plot showing differential metabolites in the ileal tissues from the C-RES-HMB group. **(D–F)** KEGG pathway enrichment analysis of ileal metabolites. RES group **(D)**, HMB group **(E)**, and RES-HMB group **(F)** compared with the C group. Differentially expressed metabolites were subjected to pathways enrichment analysis.

To determine the effects of RES/HMB addition on ileal microbial metabolism, small molecule metabolites in the ileal contents were analyzed using non-targeted metabolomics monitoring. The screening criteria were VIP value > 1 and *p*-value < 0.05 of *T*-test. A total of 156 metabolites were detected, among which 106 were up-regulated and 50 were down-regulated (Supplementary Figure S1A). The number of different metabolites between RES group, HMB group, RES-HMB group and C group was 73, 35, and 48, respectively (Figures 4A–C).

Orthogonal partial least squares discriminant (OPLS-DA) analysis was used to screen the differences of metabolites among the groups. The OPLS-DA indicated a fuzzy separation between the C and RES groups (R^2^X = 0.597, R^2^Y = 0.758, Supplementary Figure S1B), the C and HMB groups (R^2^X = 0.553, R^2^Y = 0.639, Supplementary Figure S1C), and the C and RES-HMB groups (R^2^X = 0.644, R^2^Y = 0.668, Supplementary Figure S1D), which suggested that the metabolite content of these groups may be different.

#### 3.6.2 Cluster analysis of differential metabolites in ileum

The levels of hydrocortisone 2 L-acetate, ascomycin, and ps 38:3 were significantly increased (*p* < 0.05) and the levels of thymidine 5′-triphosphate and crustecdysone were significantly decreased (*p* < 0.05) in the RES group compared with those in the C group (Supplementary Figure S2A). The HMB group significantly increased (*p* < 0.05) the levels of scopularide g and butanoic acid, while significantly decreased (*p* < 0.05) the levels of prostaglandin b2, 17-phenyltrinorprostaglandin f2.alpha. Cyclopropyl methyl amide, 7-keto-3.alpha.,12-.alpha.-dihydroxycholanic acid compared with the C group (Supplementary Figure S2B). Additionally, a significant increase in the levels of L-arginine, caftaric acid, D-mannose and retinol (*p* < 0.05) and a significant decrease in the levels of isogentisin (*p* < 0.05) were observed in the RES-HMB group versus that in the C group (Supplementary Figure S2C).

#### 3.6.3 KEGG pathway enrichment analysis of ileal metabolites

HMB supplementation mainly led to alterations in VEGF signaling pathway, arginine biosynthesis, butanoate metabolism, and protein digestion and absorption compared with the C group (Figure 4E), whereas supplementation with RES-HMB led to a greater impact on metabolic pathways including protein digestion and absorption, C-type lectin receptor signaling pathway, and vitamin digestion and absorption (Figure 4F). The metabolic pathway shared by these two test groups and the C group was protein digestion and absorption. However, there was no significant enrichment of metabolic pathways was noted in the RES addition group (Figure 4D).

## 4 Discussion

The mucosal structure of the small intestine forms the basis of its digestive and absorption functions (19), especially the villi and crypt structure of the small intestine, which are important criteria for the digestion and absorption of nutrients in ruminants (20). Crypt depth is an indicator of cell production rate, and the shallower the crypt depth of the small intestine, the stronger the secretion and absorption function of the animal intestine. In this study, dietary supplementation with HMB and RES-HMB significantly reduced the crypt depth compared with the C group. This results indicate that the simultaneous supplementation of RES-HMB has a significant positive effect on the intestinal absorption capacity of Tibetan sheep. Studies have shown that the enhancement of intestinal mucosal thickness in ruminants is conducive to improving antibacterial and anti-inflammatory capabilities, and can effectively reduce the occurrence of intestinal diseases (21). In this study, supplementation of the animal feed with RES or HMB in the diet significantly increased the mucosal thickness of the ileal of Tibetan sheep, and thus improved the disease resistance. The V/C ratio reflects the functional state of the small intestine. The larger the V/C is, the grater the absorption surface area of the intestinal epithelium is, and the stronger the digestion and absorption capacity of the intestine is (22, 23). In this study, dietary supplementation of HMB and RES-HMB significantly increased the V/C ratio compared with the C group. Our findings indicated that RES and HMB could significantly promote the growth of intestinal villi growth and improve the intestinal mucosal structure of Tibetan sheep. Our results were consistent with previous studies, in which feeding diets with RES and HMB resulted in improvement of phenotype in rumen (24) and jejunum (25) in Tibetan sheep.

The content of IgA, IgG, and IgM can reflect the level of the body’s immune system. IgA is produced by the lymphoplasma cells of the intestines in animals and serve as the first line of defense in preventing invasion by pathogenic microorganisms. In this study, dietary supplementation of RES significantly increased the content of IgA compared with the C group. Studies have shown that dietary RES can secrete IgA and inhibit the secretion of pro-inflammatory factors (26). IgG is produced and secreted by plasma cells of spleen and lymph nodes, accounting for 10–20% of all plasma proteins (27) and 70–75% of total immunoglobulins. IgG plays a role in immune functions such as antigen precipitation, bacteriophagy, and virus neutralization after antigen presentation. In this study, dietary supplementation of HMB significantly increased the content of IgG compared with the C group. This result is consistent with previous studies (28), HMB has a positive effect on improving the immune capacity of animal body. TNF-*α* is a potent inflammatory cytokine, that plays a role in cellular immunity (29). In the current study, dietary supplementation of RES-HMB combination significantly reduced the content of TNF-*α* compared with the C group. These results indicate that dietary supplementation of RES and HMB may help improve the immune status of Tibetan sheep.

Digestive enzymes are important active substances in the intestinal tracts of livestock. The activity of intestinal digestive enzymes can reflect the feeding ability of ruminants, and also directly determine the degree of intestinal absorption and utilization of nutrients, thereby contributing to the growth and development of animals. The activity of digestive enzymes in small intestine is affected by dietary intake of starch, fats and proteins. Lipase and α-amylase are mainly secreted by pancreas and small intestine. Lipases can decompose fat into fatty acids and glycerol, and α-amylase hydrolyzes starch into dextrin and maltose. Cellulase mainly breaks down the fiber in chymus. In this study, dietary supplementation of RES and RES-HMB significantly increased the activities of Lipase, Cellulase, and α-amylase. These results indicated that the supplementation of RES-HMB could enhance the activity of ileal digestive enzymes in Tibetan sheep. This enhanced effect of digestive enzymes may be attributed to the optimization of the microbial abundance in the digestive tract of Tibetan sheep, inhibition of the growth of pathogenic bacteria, increased reproduction of beneficial bacteria, and stimulation of the secretion of various digestive enzymes.

Studies have shown that intestinal flora metabolism and fermentation nutrients are the main sources of SCFAs (30). SCFAs mainly includes acetic acid, propionic acid, butyric acid, etc. About 95% of SCFAs is absorbed into the blood circulation through the colon, and then metabolized to provide 5–10% of the body’s energy (31). SCFAs can act as a direct energy substrate of host cells, stimulate the production of gastrointestinal hormones, resist the invasion of pathogenic microorganisms, and maintain the intestinal health of animals (32). Acetic acid is an important substrate for the synthesis of fat and cholesterol; propionic acid is absorbed and transported to the liver for sugar and lipid metabolism, and can inhibit cholesterol synthesis; butyric acid is used by colon epithelial cells as their main source of energy (33). In this study, the concentration of propionic acid was significantly increased in the additions of RES or HMB. This finding is consistent with results reported previously wherein RES boosted propionic acid levels in the gut of mice (34). This indicated that RES stimulated intestinal flora metabolism of Tibetan sheep to produce SCFAs, and increased the concentration of SCFAs. Bacteroidetes are responsible mainly for the synthesis of acetic acid and propionic acid in animal intestines (35).

16S rRNA analysis was used to further elucidate the effects of different dietary additives on the intestinal microflora of Tibetan sheep. In this study, different additives reduced the diversity and richness of the microbiota compared to the C group fed a standard diet. Consistent with previous studies, RES (36) and HMB (37) reduced ACE and Shannon indices. Furthermore, an increase in the relative abundance of *Proteobacteria* was noted in the RES group compared with the C group. As the largest of the bacterial phylum, an increase in the abundance of *Proteobacteria* is often associated with intestinal inflammation, and it plays an important role in maintaining the anaerobic environment of the intestine (38). Besides, the relative abundance of *Bacteroidota* and *Firmicutes* were also increased in the HMB group. *Bacteroidetes* are key microbes involved in the regulation of the body’s immune system, and they secrete metabolites that help maintain the stability of the immune system. These metabolites are the bulk of the production of SCFAs in the gut, mainly in the form of acetate and propionate, which are important for maintaining intestinal homeostasis (39). Notably, the relative abundance of some species in *Firmicutes* such as *Brevibacillus*, *Clostridium sensu stricto 3*, and *Eubacterium hallii group* were also increased in the RES-HMB group. *Firmicutes* play a key role in host nutrition and metabolism through the synthesis of SCFAs, and have a strong ability of immune regulation and inhibition of opportunistic pathogen invasion (40). It has been reported that the supplementation of *Brevibacillus laterosporus* S62-9 (10^6^ colony forming units/g diet) can improve the composition of intestinal microbial community of broilers, thereby enhancing their growth performance and immunity (41). *Clostridium* is one of the largest bacterial genera, and many clostridium bacteria form butyrate as their main fermentation product (42). *Clostridium* have positive or negative effects on the body (43). Among them, *Clostridium tetani*, *Clostridium botulinum* and *Clostridium difficile* can negatively impact on animal health (44), whereas other species of *Clostridium* can help with the digestion of complex organic matter in the gastrointestinal tract (45). Therefore, we speculated that *Clostridium sensu stricto 3* may be related to the level changes of intestinal SCFAs and show a negative correlation, and the specific mechanism needs to be further studied.

Changes in the gut flora are often accompanied by changes in metabolites (41). In the present study, the content of L-arginine, which is related to arginine biosynthesis, was increased by HMB. As one of the most commonly used amino acids, L-arginine can be used as a precursor for a variety of compounds (46). For example, arginine is a common precursor for the synthesis of NO and polyamines, which are essential for placental development and growth in mammals (47). It has also been found that dietary arginine supplementation can improve meat quality, antioxidant defense, and growth performance of broilers (48). In this study, the level of butanoic acid related to butanoate metabolism was increased by HMB. Butanoic acid is an important, multifaceted anti-inflammatory agent that helps improve immune tolerance, increase intestinal T-regulatory cells, and regulate macrophages (49). It was found that the digestibility of dry matter, protein, crude fat and calcium of calves increased significantly upon supplementation of the diet with sodium butyrate (50). Sodium butyrate supplementation also enhances digestibility in the intestines of animals. Furthermore, the levels of L-arginine and butanoic acid associated with the pathway of protein digestion and absorption were increased in the RES-HMB group. Moreover, the levels of D-mannose related to C-type lectin receptor signaling pathway was increased by RES-HMB. D-mannose can enhance intestinal barrier by producing SCFAs through fermentation. Some probiotics (*Bifidobacterium*, *Lactobacillus*, etc.) in the intestine ferments oligosaccharides that are difficult to digest and produce SCFAs, stimulating intestinal bacteria to secrete mucus and produce antimicrobial peptides, and increasing intestinal mucosal immune response (51). In addition, the abundance of retinol related to the vitamin digestion and absorption was increased in RES-HMB supplemented sheep. VA (retinol) has long been regarded as an essential nutrient for maintaining normal immunity, and it is involved in humoral and cellular immunity, and plays an important role in T cell proliferation and antibody synthesis (52). Retinol can inhibit colon SCFAs production in the colons of the offspring by reducing the relative abundance of SCFAs producing gut microbes during early pregnancy in rats (53). Therefore, the changes in vitamin and protein metabolism that were noted in this study may have a positive effect on the growth and development of Tibetan sheep.

## 5 Conclusion

In conclusion, our findings revealed that dietary supplementation with RES and HMB could affect the composition of the intestinal microflora and metabolites of Tibetan sheep, and promote the abundance of *Brevibacillus*, *Clostridium sensu stricto 3*, and *Eubacterium hallii group.* Furthermore, an increase in the levels of differential metabolites including L-arginine, butanoic acid, D-mannose and retinol was noted. These changes in intestinal flora may contribute to improving the digestive enzyme activity and SCFAs content of Tibetan sheep, thereby improving their growth performance, immune level and digestive capacity. Collectively, these findings provide insights into detailed strategies to improve the ileum health of Tibetan sheep.

## Data Availability

The datasets presented in this study can be found in online repositories. The names of the repository/repositories and accession number(s) can be found in the article/Supplementary material.

## Supplementary material

Figure S1 Differences in ileal metabolome. **(A)** Number of different metabolites between control and experimental groups. **(B–D)** Orthogonal partial least square method (OPLS-DA) analysis between RES group **(B)**, HMB group **(C)**, RES-HMB group **(D)**, and C group.

Figure S2 Cluster analysis of differential metabolites in ileum. Cluster analysis of differential metabolites between RES group **(A)**, HMB group **(B)**, RES-HMB group **(C)**, and C group.
